# Wnt signaling in orofacial clefts: crosstalk, pathogenesis and models

**DOI:** 10.1242/dmm.037051

**Published:** 2019-02-04

**Authors:** Kurt Reynolds, Priyanka Kumari, Lessly Sepulveda Rincon, Ran Gu, Yu Ji, Santosh Kumar, Chengji J. Zhou

**Affiliations:** 1 Department of Biochemistry and Molecular Medicine, University of California at Davis, School of Medicine, Sacramento, CA 95817, USA; 2 Institute for Pediatric Regenerative Medicine of Shriners Hospitals for Children, University of California at Davis, School of Medicine, Sacramento, CA 95817, USA; 3 Biochemistry, Molecular, Cellular, and Developmental Biology (BMCDB) Graduate Group, University of California, Davis, CA 95616, USA

**Keywords:** Orofacial clefts, Cleft lip, Cleft palate, Wnt, Bmp, Fgf, Tgfβ, Shh, Retinoic acid, Epigenetics, Crosstalk

## Abstract

Diverse signaling cues and attendant proteins work together during organogenesis, including craniofacial development. Lip and palate formation starts as early as the fourth week of gestation in humans or embryonic day 9.5 in mice. Disruptions in these early events may cause serious consequences, such as orofacial clefts, mainly cleft lip and/or cleft palate. Morphogenetic Wnt signaling, along with other signaling pathways and transcription regulation mechanisms, plays crucial roles during embryonic development, yet the signaling mechanisms and interactions in lip and palate formation and fusion remain poorly understood. Various Wnt signaling and related genes have been associated with orofacial clefts. This Review discusses the role of Wnt signaling and its crosstalk with cell adhesion molecules, transcription factors, epigenetic regulators and other morphogenetic signaling pathways, including the Bmp, Fgf, Tgfβ, Shh and retinoic acid pathways, in orofacial clefts in humans and animal models, which may provide a better understanding of these disorders and could be applied towards prevention and treatments.

## Abstract (precis)

**Summary:** Investigation of the crosstalk between the Wnt and other signaling pathways will improve understanding of orofacial cleft development and provide opportunities for treatment and prevention.

## Introduction

Orofacial clefts, mainly cleft lip and/or cleft palate, are among the commonest structural birth defects (Tolarova and Cervenka, 1998; Mossey et al., 2009; Shkoukani et al., 2014; Roosenboom et al., 2015; Kousa and Schutte, 2016). The occurrence of orofacial clefts varies with geographic and ethnic background and with socioeconomic status, with an average rate of 1 in 700 newborns or a range of 0.5-2.6 per 1000 live births (Vanderas, 1987; Croen et al., 1998; Carmichael et al., 2003; Panamonta et al., 2015). Children born with orofacial clefts have severe feeding problems, speech difficulties, frequent middle ear infections and dental defects (Mossey et al., 2009). The long-term and multidisciplinary treatments for these problems are a heavy burden for patients and the healthcare system. Orofacial clefts can either be syndromic or non-syndromic, sporadic or familial (see Glossary, Box 1), and their etiology involves a combination of genetic and environmental risk factors (Tolarova and Cervenka, 1998; Mossey et al., 2009). To date, more than 100 genes have been associated with orofacial clefts (Gritli-Linde, 2007; Juriloff and Harris, 2008; Bush and Jiang, 2012; Iwata et al., 2012), but the underlying mechanisms of these associations remain poorly understood. Mutant mouse models have provided a powerful tool to examine the roles of various genes in contributing to orofacial clefts.

**Box 1. Glossary**

**C6 motif:** the six-amino-acid C-terminal domain of Axin proteins. It is implicated in JNK activation, but has no effect on Wnt signaling.

**Epithelial-mesenchymal transition (EMT):** the induction of adhesive epithelial cells to become migratory and proliferative cells during developmental processes, including in palatogenesis.

**Goltz-Gorlin syndrome:** a rare genetic disorder, also known as focal dermal hypoplasia (FDH), characterized by distinctive skin abnormalities, including CLP in some cases, and other defects that affect eyes, teeth, and the skeletal, urinary, gastrointestinal, cardiovascular and central nervous systems. Mutations in PORCN, an upstream regulator of Wnt signaling, are associated with FDH.

**Maxillary prominences:** a pair of developmental structures at the lateral edges of the oral cavity that give rise to the upper jaw elements, including the maxillary bone.

**Medial edge epithelium (MEE):** the distalmost edge of the palatal epithelium that surrounds the proliferating mesenchyme. On each palatal shelf, this layer will meet and fuse during secondary palatogenesis.

**Midline epithelial seam (MES):** the layer of epithelial cells that separates the two lateral pools of the mesenchyme after palatal shelf fusion. MES cells undergo apoptosis to allow the formation of a continuous mesenchyme layer across the secondary palate.

**Nasal prominences:** two pairs of medial and lateral extensions derived from the unpaired frontonasal prominence during early craniofacial development, which fuse on either side with the maxillary prominences to form the primary palate and nostrils, and separate the nasal cavity from the oral cavity.

**Neurulation:** a stage in vertebrate embryogenesis in which the neural plate folds to form the neural tube.

**Palatal shelf:** a pair of palatal structures elongated from the maxillary prominences between the nasal prominence and the first branchial arch/mandibular process, which eventually fuse to separate the oral and nasal cavities.

**Primary palate:** a rostralmost palatal structure formed by the fusion of the nasal and maxillary prominences to separate the nasal pits from the oral cavity.

**Regulator of G protein signaling (RGS) domain:** a motif required for the protein's activity in accelerating the GTPase activity of G-proteins. The RGS domain in Axin proteins is required for binding APC in Wnt signaling.

**Robinow syndrome:** congenital syndrome characterized by craniofacial, skeletal and urogenital defects, which frequently includes orofacial clefts and has been associated with mutations in noncanonical Wnt signaling genes, including *WNT5A* and *ROR2*.

**Rugae:** the series of ridges produced by folding of the anterior wall of the palate behind the incisive papillae.

**Secondary palate:** a roof structure of the oral cavity that arises from the fusion of the left and right palatal shelves posterior to the primary palate.

**Sporadic or familial CLP:** occurrence of CLP within families or close relatives is referred to as familial CLP, whereas appearance of the phenotype without apparent genetic predisposition is termed sporadic CLP.

**Submucous cleft palate:** a form of cleft palate in which the two palatal shelves incompletely fuse such that the oral and nasal cavities are separated from each other by soft tissue, but a continuous layer of bone has not developed across the midline.

**Syndromic or non-syndromic CLP:** CLP patients carrying additional dysmorphic or clinical features are syndromic; non-syndromic CLP is not associated with other phenotypes.

**Tetra-amelia syndrome:** a congenital syndrome characterized by limb malformation, often coupled with craniofacial and urogenital defects, associated with *WNT3* mutations.

**Unilateral and bilateral CLP:** a cleft can occur either at one (unilateral) or both sides (bilateral) of the face.

**Van der Woude syndrome:** a congenital syndrome characterized by craniofacial, limb, and cardiac defects, associated with mutations in the transcription factors downstream of canonical Wnt signaling.

Murine and human facial formation follow a similar developmental trajectory, and facial structures arise from several primordial tissues as described below (Francis-West et al., 1998; Schutte and Murray, 1999; Jiang et al., 2006; Szabo-Rogers et al., 2010; Suzuki et al., 2016). Facial primordia begin to form as early as the fourth week of gestation in humans or embryonic day (E) 9.5 in mice, following the migration of cranial neural crest cells into the frontonasal prominence, paired maxillary prominences (Box 1) and paired mandibular prominences (Cordero et al., 2011). By the fifth week, the medial and lateral nasal prominences (Box 1) outgrow rapidly on either side of the nasal pit. At the ventral junction region, these nasal prominences will subsequently fuse with the maxillary prominence to establish the upper jaw structures, including the upper lip, primary palate (Box 1) and nose. Disruption of any of these early craniofaciogenic processes may result in cleft lip with or without cleft palate (CLP). Secondary palate (Box 1) formation is a multifaceted process involving a shift in growth orientation by the palatal shelves (Box 1) (Lough et al., 2017).

In mice, the palatal shelves first emerge from the maxillary prominences at E11.5 and continue to proliferate, elongating ventrally between E12 and E14 (Bush and Jiang, 2012). The elongating palatal shelves consist of mesenchymal tissue with an external epithelial layer. Epithelial-mesenchymal interactions (EMIs) allow communication between the two layers and are important for cell growth and differentiation during many craniofacial developmental processes, including facilitating epithelial-mesenchymal transition (EMT; Box 1) within the palatal shelves during palatogenesis (Sun et al., 1998; Lan and Jiang, 2009; Levi et al., 2011; Santosh and Jones, 2014). The palatal shelves then elevate and continue to grow horizontally toward the midline, which entails significant extracellular matrix remodeling (Bush and Jiang, 2012), until they fuse along the medial edge epithelium (MEE; Box 1) at E14.5-E15. The palatal shelves at the midline fuse both anteriorly and posteriorly from the initial point of contact in a zipper-like manner to form a midline epithelial seam (MES; Box 1). Disintegration of the MES, which may involve apoptosis, EMT and cell migration, is required to establish palatal confluence (Bush and Jiang, 2012). At E15.5-E16.5, the palatal shelves fuse with the nasal septum and the primary palate, separating the nasal and oral cavities, which are required for breathing and feeding after birth (Gritli-Linde, 2007). Disruptions during any stage of palatogenesis can result in a cleft palate (Dixon et al., 2011). Although the mechanisms that drive palatogenesis are believed to be conserved among mammals, differences in the morphological structures, and in the interactions that occur during palatal closure, exist between species (Yu et al., 2017). An extensive list of different mouse models for cleft lip and/or cleft palate has been previously reviewed elsewhere (Gritli-Linde, 2007; Gritli-Linde, 2008; Juriloff and Harris, 2008; Funato et al., 2015). However, mutations in specific genes do not always produce the same phenotype in humans and mouse models (Gritli-Linde, 2008).

**Fig. 1.**
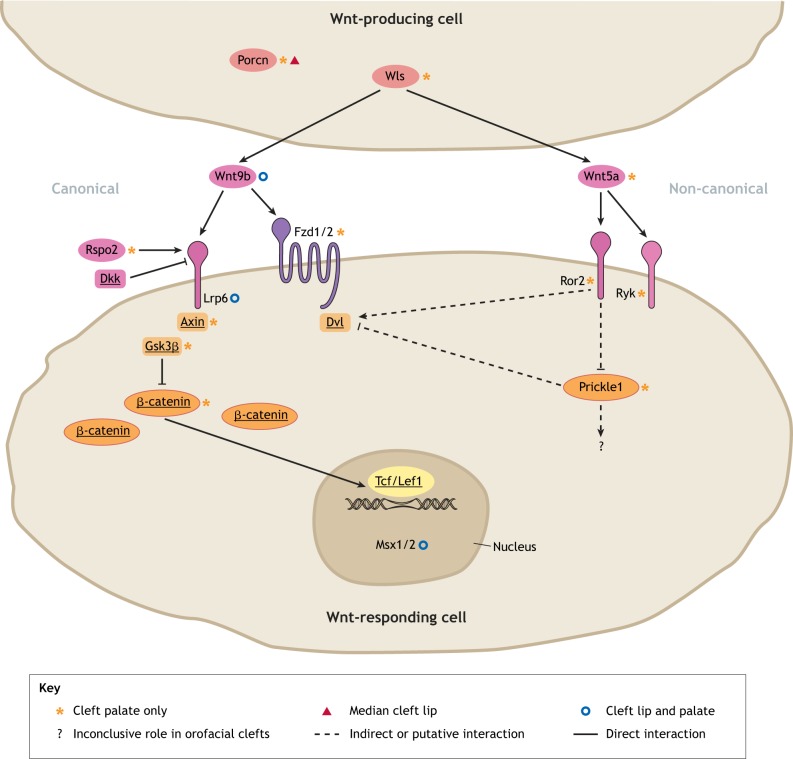
**Key components and potential pharmacological targets of Wnt signaling implicated in orofacial clefts.** Wnt proteins are lipid modified by Porcn and secreted in the extracellular matrix by Wls from the Wnt-producing cells. Wnt9b and Wnt5a are representative orofacial cleft-associated ligands in the canonical and non-canonical Wnt signaling pathways, respectively. In the canonical pathway, Wnt9b may bind to receptor Fzd1/Fzd2 and co-receptor Lrp6, which respectively recruit Dvl and Axin proteins, disrupting the β-catenin destruction complex that includes the glycogen synthase kinase Gsk3β in the Wnt-responding cells. This leads to intracellular accumulation of β-catenin and its translocation to the nucleus, where, together with the Tcf/Lef1 transcription factors, it regulates the expression of downstream target genes that are critical during lip and palate formation, such as *Msx1* and *Msx2*. The Lrp6 co-receptor can be regulated positively by Rspo2 and negatively by Dkk proteins. In the non-canonical pathway, Wnt5a binds to co-receptor Ror2 and/or Ryk to suppress Prickle1, which in turn activates Dvl proteins (involved in both canonical and non-canonical pathways) and facilitates the cytoskeletal rearrangements during palatogenesis. It is unclear whether Wnt5a also binds to Fzd/Lrp proteins to regulate canonical Wnt signaling during orofacial development. The key components of the signaling cascade that are underlined in the diagram could be targeted by small molecules to modulate Wnt signaling. Orofacial cleft-associated signaling molecules are marked with symbols according to their resulting phenotype (see figure key).

Wingless-type MMTV integration site (Wnt) signaling is required for body axis patterning, cell fate specification, cell proliferation and cell migration during embryonic development (Kimura-Yoshida et al., 2005; Komiya and Habas, 2008; Basson, 2012; Clevers and Nusse, 2012; Perrimon et al., 2012; Hikasa and Sokol, 2013; Clevers et al., 2014; Nusse and Clevers, 2017). Wnt signaling (see Box 2) is active in most tissues during craniofacial development (Mani et al., 2010), and includes multiple distinct pathways that are activated by the binding of the secreted Wnt ligand proteins to a complex receptor system. Wnts bind to the frizzled (Fzd) receptors along with the co-receptors, such as members of the low-density lipoprotein receptor-related protein (Lrp) or receptor tyrosine kinase-like orphan receptor (Ror) families, at the surface of the Wnt-responding cells (Fig. 1, Box 2). The ligand-receptor complex interacts with cytoplasmic proteins, such as the axis inhibition (Axin) and disheveled (Dvl) proteins, to trigger intracellular signaling (Wallingford and Habas, 2005; Niehrs, 2012; Stamos and Weis, 2013; Bernatik et al., 2011) (Fig. 1, Box 2). Wnt pathways are broadly classified as β-catenin-dependent canonical and β-catenin-independent non-canonical pathways, such as the planar cell polarity (PCP) pathway (Box 2) and the Wnt/Ca^2+^ pathway (Komiya and Habas, 2008; Gao and Chen, 2010). This Review discusses the role of Wnt signaling and its crosstalk with other signaling pathways in orofacial cleft etiology and related developmental processes, which may provide a better understanding of basic mechanisms and future translational applications.

**Box 2. Wnt signaling**

Wnts are secreted lipid-modified signaling proteins that are evolutionally conserved and play vital roles in development, homeostasis and disease. Nineteen Wnt ligand proteins encoded by respective genes in mammalian genomes act through a variety of receptors and co-receptors, including ten seven-transmembrane frizzled (Fzd) receptors, two single-transmembrane co-receptors Lrp5/6, and the receptor tyrosine kinase-like receptors Ror1/2 and Ryk.

In the Wnt-producing cells, the nascent Wnt proteins are palmitoylated by porcupine O-acyltransferase (Porcn), followed by their secretion in the extracellular matrix via Wnt ligand secretion mediator (Wls; Fig. 1). Wnt signaling is initiated when a secreted Wnt ligand binds to a Fzd receptor along with a Lrp co-receptor in the canonical pathway, or to a tyrosine kinase-like Ror or Ryk receptor in the non-canonical pathway. The ligand-receptor interaction at the surface of the Wnt-responding cell is modulated by various positive or negative regulatory factors and is transmitted through numerous intracelluar molecules. Three major signaling pathways have been demonstrated downstream of the initial Wnt ligand-receptor interaction: the canonical Wnt/β-catenin signaling pathway, the non-canonical planar cell polarity (PCP) pathway, and the Wnt/Ca^2+^ pathway, which is less understood.

Canonical Wnt/β-catenin pathway: when Wnts are absent, intracellular β-catenin is constantly phosphorylated for degradation by the glycogen synthase kinase Gsk3β in the β-catenin destruction complex, which also includes the tumor suppressing Axin proteins and adenomatous polyposis coli (APC), the casein kinase CK1, the protein phosphatase 2A (PP2A) and the E3-ubiquitin ligase β-TrCP. Upon the binding of a Wnt ligand to a Fzd receptor, the Fzd recruits a Dvl cytoplasmic phosphoprotein and a Lrp co-receptor recruits an Axin, which inhibits the destruction complex. This stabilizes cytoplasmic β-catenin, resulting in its accumulation and translocation into the nucleus. There it binds to Tcf/Lef1 transcription factors to regulate the transcriptional activation of critical Wnt target genes in various cells/tissues, such as the orofacial cleft-associated genes *Msx1/Msx2* in orofacial primordia (Fig. 1).

Non-canonical PCP pathway: The binding of a Wnt ligand to Ror or Ryk receptors promotes the interaction of a Dvl with disheveled-associated activator of morphogenesis 1 (Daam1), which activates several downstream GTPases, including the Rac proteins and ras homolog family member A (RhoA). This results in the restructuring of actin to change cell shape, polarity and movement. Dvl can also activate phospholipase C to generate inositol triphosphate, which activates the release of Ca^2+^ to trigger a number of downstream effects, such as cell migration and proliferation.

## WNT signaling genes associated with orofacial clefts in humans

Both syndromic and non-syndromic orofacial clefts have been attributed to mutations of various WNT signaling component genes (Table 1). Nascent WNT proteins are lipid modified by the enzyme porcupine O-acyltransferase (PORCN) within the endoplasmic reticulum of the WNT-producing cell and subsequently transported by WNT ligand secretion mediator (WLS, also known as *GPR177*) through the Golgi apparatus to the cell surface for secretion (Port and Basler, 2010; Barrott et al., 2011) (Fig. 1, Table 1, Box 2). An extensive number of mutations throughout the coding region and large gene rearrangements of *PORCN* have been identified in focal dermal hypoplasia or Goltz–Gorlin syndrome (Box 1), which includes orofacial clefts (Table 1) (Lombardi et al., 2011). However, a role for PORCN in non-syndromic cleft lip and palate (NSCLP), or a role for WLS in human orofacial clefts, has not been demonstrated. A homozygous nonsense mutation in *WNT3* has been correlated with orofacial clefts and tetra–amelia syndrome (Box 1) (Niemann et al., 2004). Meanwhile, multiple non-coding single-nucleotide polymorphisms (SNPs) in *WNT3* have been associated with NSCLP in a wide range of populations, including Latin American, European and Chinese (Chiquet et al., 2008; Nikopensius et al., 2010; Nikopensius et al., 2011; Mostowska et al., 2012; Lu et al., 2015). Yet, in some populations, such as Caucasian Brazilian, the relationship between *WNT3* variants and NSCLP remains unclear (Fontoura et al., 2015; Machado et al., 2016). Intriguingly, Nikopensius et al. (2010) reported a potential epistatic interaction between *WNT3* and collagen, type II, alpha 1 (*COL2A1*), an important gene in the production of collagen. Mutations in either gene are associated with NSCLP (Nikopensius et al., 2010; Nikopensius et al., 2011), and *COL2A1* mutations also cause Stickler Syndrome, which frequently includes a cleft palate only (CPO) phenotype (Hoornaert et al., 2010), suggesting a relationship between canonical WNT signaling and the extracellular matrix during palatogenesis. *WNT3* is clustered at 17q21.31-17q21.32 with *WNT9B*, variants of which were also associated with a predisposition to NSCLP (Nikopensius et al., 2011; Fontoura et al., 2015). Several SNPs near the *WNT6-WNT10A* cluster at the 2q35 region of chromosome 2 were associated with either CLP or CPO (Beaty et al., 2006), while more recently, a missense mutation within *WNT10A* was identified in a Chinese NSCLP cohort (Feng et al., 2014). A *WNT7A* variant containing a missense SNP was identified as a contributor to NSCLP in several heterozygous members of a multi-case family (Pengelly et al., 2016).

**Table 1.**
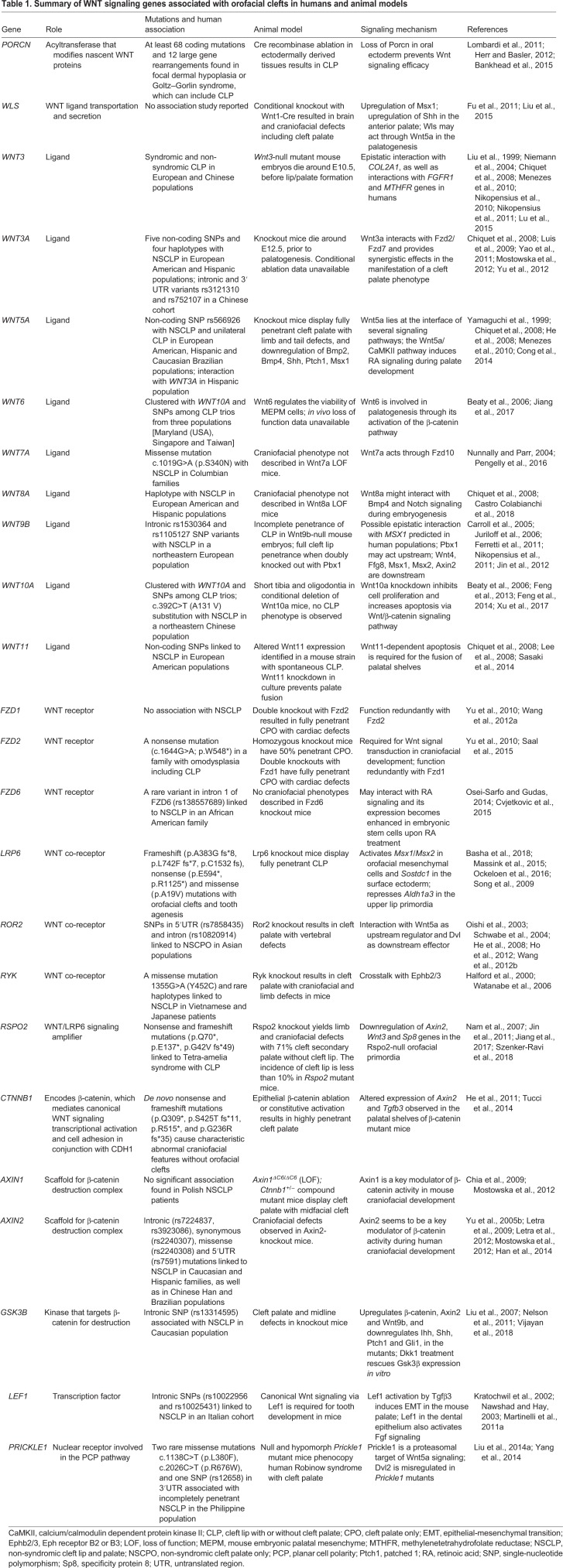
**Summary of WNT signaling genes associated with orofacial clefts in humans and animal models**

Intronic and 3′UTR SNPs in the prototypical canonical WNT ligand gene *WNT3A* have also been identified in patients with NSCLP within European American, Hispanic and Chinese populations, with homozygosity presenting an increased risk over heterozygosity for each allele identified in the Chinese cohort (Chiquet et al., 2008; Yao et al., 2011) (Table 1). Association analyses within the European American cohort also implicated *WNT11*, while an association between SNPs in the non-coding regions downstream of *WNT3A* and the non-canonical *WNT5A* was reported in the Hispanic population (Chiquet et al., 2008) (Table 1). An intronic *WNT5A* SNP was associated with unilateral CLP (Box 1) with marginal significance in a Caucasian Brazilian population (Menezes et al., 2010). Robinow syndrome (Box 1), which frequently includes cleft palate, is associated with mutations in *WNT5A* (Person et al., 2010), along with mutations in the co-receptor gene receptor tyrosine kinase-like orphan receptor 2 (*ROR2*) (Afzal et al., 2000; van Bokhoven et al., 2000) and the signal transducer *DVL1* (Bunn et al., 2015; White et al., 2015), indicating the importance of a non-canonical WNT5A/ROR2/DVL1 signaling cascade in human palatogenesis. A missense mutation and rare haplotypes of another non-canonical co-receptor gene, receptor-like tyrosine kinase (*RYK*), have also been linked to NSCLP in Vietnamese and Japanese patients (Watanabe et al., 2006). Additionally, several non-coding variants and one missense SNP of *PRICKLE1* have demonstrated association with non-canonical WNT signaling and human NSCLP (Yang et al., 2014).

Analysis of an African American family with 11 members displaying NSCLP identified a variant of the WNT receptor gene *FZD6* with an intronic mutation that creates a protein-binding site, resulting in decreased expression and contributing to CLP (Cvjetkovic et al., 2015). Among other FZD genes, a nonsense mutation of *FZD2* was identified in a family with omodysplasia that includes CLP (Saal et al., 2015). By contrast, frameshift, nonsense and missense mutations in the WNT co-receptor gene *LRP6* have been associated with orofacial clefts and tooth agenesis (Basha et al., 2018; Ockeloen et al., 2016), suggesting that deficient LRP6-mediated canonical WNT signaling has a crucial role in CLP pathogenesis. However, *de novo* nonsense and frameshift mutations in the key canonical WNT signaling mediator gene catenin beta 1 (*CTNNB1*; encoding β-catenin) were linked with abnormal craniofacial features, but not with orofacial clefts (Tucci et al., 2014). Conversely, analysis of variants of the β-catenin destruction complex genes *AXIN2* and glycogen synthase kinase 3 beta (*GSK3B*) in NSCLP families across multiple populations identified intronic SNPs that contribute to orofacial clefts (Letra et al., 2009; Letra et al., 2012; Vijayan et al., 2018) (Table 1), suggesting that excessive WNT/β-catenin signaling also contributes to CLP pathogenesis. Nevertheless, gene association studies in humans with orofacial clefts have proven challenging, complicated by the fact that the same variants can be associated with orofacial clefts in one population but not in others. Therefore, animal models, especially the mutant mouse model, have a crucial role in investigating the genetic mechanisms of orofacial clefts in mammals.

## Wnt signaling genes as the cause of orofacial clefts in animal models

Mutations in various Wnt signaling genes cause orofacial clefts in animal models (He and Chen, 2012) (Table 1). The following discussion highlights animal models of orofacial clefts involving mutations in both canonical and non-canonical Wnt signaling components, from ligand secretion through signal transduction, focusing predominantly on mouse models.

### Mouse models with mutations in regulatory genes upstream of Wnt

Absence of Porcn or Wls from Wnt-producing cells (Fig. 1) results in Wnt protein retention, which leads to Wnt signaling failure (Barrott et al., 2011). Conditional ablation of Porcn in neural crest cells results in defective facial formation in mice, including CLP (Bankhead et al., 2015) (Fig. 1). Previous studies suggest that Wls is required for Wnt/β–catenin signaling during craniofacial development (Fu et al., 2011). Facial ectodermal/epithelial ablation of either β-catenin or Wls arrests the formation of orofacial primordia (Wang et al., 2011; Zhu et al., 2016), and conditional knockout of Wls in craniofacial neural crest cells with a Wnt1-driven Cre recombinase causes cleft palate (Liu et al., 2015) (Table 1). Additionally, Rspo2, a member of the R-spondin family, is a well-known enhancer of canonical Wnt signaling (Fig. 1). Rspo2 loss-of-function mice exhibit cleft palate with a partially penetrant cleft lip, along with mandibular hypoplasia and maxillary and mandibular skeletal deformation, which are caused by defective patterning and morphogenesis of the first pharyngeal arch due to altered EMI (Yamada et al., 2009; Jin et al., 2011). However, cleft palate in Rspo2-null mice is likely caused by delayed palatal shelf (Box 1) elevation, a possible secondary effect of aberrant mandible and tongue morphogenesis (Jin et al., 2011). Nevertheless, Wnt signaling may mediate Tgfβ signaling to regulate EMI in muscle development of the soft palate (Iwata et al., 2014).

### Mouse models with mutations in canonical Wnt signaling genes

Among the 19 Wnt ligands, Wnt2, Wnt2b, Wnt4, Wnt5a, Wnt5b, Wnt6, Wnt7b, Wnt9a, Wnt10a, Wnt10b, Wnt11 and Wnt16 are expressed in the palatal primordia during palatogenesis (Warner et al., 2009). Five Wnts appear to be temporally regulated in embryonic palatal tissue, showing more than 2.0-fold changes in expression levels, either between E12.5 and E13.5, or between E13.5 and E14.5. Of these five ligands, Wnt4, Wnt10a and Wnt10b are expressed in epithelial tissues, while Wnt2 and Wnt16 are expressed in the mesenchyme (Warner et al., 2009). However, the roles of these temporarily expressed Wnts in palatogenesis remain unclear. By contrast, Wnt6 has been demonstrated to play a role in palatal shelf elongation and elevation through the activation of the β-catenin pathway, promoting cell proliferation in the palatal mesenchyme (Jiang et al., 2017) (Table 1).

Wnt9b might activate the canonical Wnt signaling pathway during midfacial development (Lan et al., 2006). Wnt9b-null mice die perinatally, exhibiting incompletely penetrant CLP (Carroll et al., 2005; Juriloff et al., 2006; Ferretti et al., 2011) (Table 1, Fig. 1), while ablation of Wnt9b in the facial ectoderm also causes CLP (Jin et al., 2012). These findings suggest a key role of facial ectodermal and epithelial Wnt/β-catenin signaling in primary lip and palate formation and fusion. In addition, Wnt3 may also regulate midfacial development, as well as lip fusion, through the canonical Wnt pathway, with both Wnt9b and Wnt3 playing distinct roles during midfacial morphogenesis (Lan et al., 2006). Wnt3-null embryos do not survive beyond E10.5, while morphological differences from wild-type embryos become apparent from E6.5 onward (Liu et al., 1999). *In vitro* experiments further suggest that Wnt3 and Wnt9b may activate canonical Wnt signaling during palatogenesis through the receptors Fzd1 and Fzd2 (Lan et al., 2006; Yu et al., 2010). Palatal shelves fail to close in doubly homozygous Fzd1 and Fzd2 knockout mice with complete penetrance (Yu et al., 2010) (Table 1, Fig. 1), while Fzd7 is highly redundant with Fzd2 during palatogenesis (Yu et al., 2012). Canonical Wnt signaling through the co-receptor Lrp6 plays an indispensable role in primary lip and palate formation and fusion (Song et al., 2009; Zhou et al., 2010). *Lrp6*-deficient mutant mouse embryos exhibit fully penetrant CLP as a consequence of diminished Wnt signaling and disrupted expression of downstream target genes in the orofacial primordia (Song et al., 2009) (Fig. 1).

While conditional loss of function of β-catenin in palatal epithelial cells leads to cleft palate, conditional gain of function of β-catenin in the epithelium also leads to cleft palate and aberrant fusion between the palate shelf and mandible (He et al., 2011), suggesting crucial roles of epithelial Wnt signaling in palatal shelf fusion. Moreover, homozygous knockout of *Gsk3b*, which encodes a β-catenin-degrading enzyme in the canonical Wnt signaling pathway, results in mice displaying full cleft palate (Liu et al., 2007) (Table 1, Fig. 1), suggesting that excessive β-catenin signaling also causes cleft palate in these mouse models.

Axin1 is another component of the β-catenin destruction complex and therefore a negative regulator of Wnt signaling (Fig. 1). Early embryonic lethality is observed in homozygous *Axin1* mutant mouse embryos carrying alleles with deletions in either the regulator of G protein signaling (RGS) domain (Box 1) or the C6 motif (Box 1) that encodes the six C-terminal amino acids (Axin1^ΔC6^) (Chia et al., 2009). Intriguingly, many mouse embryos with compound mutant alleles of *Axin1^ΔC6/ΔC6^* and *Ctnnb1^+/−^* can survive to term but develop craniofacial defects, including CLP (Chia et al., 2009) (Table 1, Fig. 1). This suggests that diminished Wnt/β-catenin signaling can partially rescue the early lethality that is likely caused by excessive β-catenin signaling, but it cannot rescue the CLP phenotype that may be caused by both excessive β-catenin and defective JNK signaling (Chia et al., 2009). Together, these findings highlight the importance of appropriate spatiotemporal control of Wnt/β-catenin signaling and the complexity of the regulatory processes in lip and palate development.

### Mouse models with mutations in non-canonical Wnt signaling genes

Wnt5a acts through the non-canonical Wnt pathway to alter directional cell movements (Liu et al., 2015). Wnt5a-null mouse embryos exhibit cleft palate (Table 1, Fig. 1), along with other phenotypes, such as defective outgrowth of the snout, tongue, mandible, limb, tail and other skeletal defects, leading to perinatal lethality (Yamaguchi et al., 1999; Li et al., 2002; Yang et al., 2003; Cervantes et al., 2009; Tai et al., 2009; Buttler et al., 2013; Okamoto et al., 2014). Wnt5a plays a key role in the migration of mesenchymal cells during palatogenesis (Xiao et al., 2005; He et al., 2008), possibly acting through Ror2, which is expressed in the mesenchyme of the secondary palate (Schwabe et al., 2004). Studies suggested that Wnt5a binds to the cysteine-rich domain of Ror2 to activate the non-canonical Wnt pathway, interacting both physically and functionally (Oishi et al., 2003). In mesenchymal cell culture, cell migration seems to be driven by the Wnt5a-Ror2-Kif26b signaling cascade (Susman et al., 2017), further suggesting the significance of this non-canonical Wnt signaling cascade in palatogenesis. Furthermore, phosphorylation of the Wnt signal transducer Dvl2 seems to be triggered by the Wnt5a-Ror2 pathway, and Dvl2 may be the molecular switch that allows Wnt5a to activate both non-canonical and canonical Wnt pathways (Ho et al., 2012).

Ror2 knockout mice display craniofacial defects, including cleft palate, further implicating this cascade in the etiology of non-canonical Wnt-signaling-caused orofacial clefts (Schwabe et al., 2004). It has also been suggested that the Ryk receptor may interact with Ror2 to bind Wnt5a (Oishi et al., 2003), and mutations in Ryk also cause cleft palate in mice (Halford et al., 2000) (Table 1, Fig. 1). In addition, ablation of the non-canonical Wnt signaling molecule Prickle1 causes cleft palate and limb defects (Yang et al., 2014) (Table 1, Fig. 1), which are similar to those of *Wnt5a* mutants (He et al., 2008). However, *Prickle1* mutants present less severe limb defects than *Wnt5a* mutants, implying that the transduction of Wnt5a signaling might not act through Prickle1 alone. Similarly to *Wnt5a* mutants, Prickle1 knockout mice present with improper sonic hedgehog (Shh) expression during palatogenesis (Yang et al., 2014). Furthermore, Prickle1 has been shown to act downstream of Wnt5a and interact with Dvl2, and *Prickle1* mutants display characteristics that resemble Robinow syndrome (Liu et al., 2014a) (Fig. 1). Thus, a signaling cascade of Wnt5a-Ror2-Prickle1/Dvl2 might be crucial for proper tissue growth and morphogenesis during palatogenesis in mice.

### Zebrafish models

Although mouse models have vastly contributed to our understanding of Wnt signaling in palatogenesis, other models, such as the zebrafish, provide unique insight into craniofacial formation and the basic requirements for palate formation (Duncan et al., 2017). Canonical Wnt signaling through Lrp5 is required for appropriate cranial neural crest cell migration, but not their induction, and for craniofacial morphogenesis in zebrafish (Willems et al., 2015). Wnt9a is expressed in the zebrafish pharyngeal arch, implicating its role during craniofacial development (Curtin et al., 2011). Interestingly, Wnt9a has been shown to play a role in palatogenesis in fish, but not in mammals (Dougherty et al., 2013; Rochard et al., 2016), suggesting taxon-specific Wnt signaling functions in palatogenesis. Wnt5b is thought to assume a similar craniofacial role in zebrafish that Wnt5a plays in mammals (Topczewski et al., 2011). Non-canonical Wnt signaling mediated by epithelial Wnt5b and Wnt9b was demonstrated to stimulate the PCP pathway in chondrocytes, facilitated by Secreted frizzled-related protein 3 (Sfrp3, also known as Frzb) and Glypican 4 (Gpc4) activity during palate extension (Rochard et al., 2016). Additionally, morpholino-based knockdown of Wnt3a and Tubulointerstitial nephritis antigen-like 1 (Tinagl1), a Wnt-interacting extracellular matrix protein, results in defects of the pharyngeal arch and ethmoid plate, which corresponds to the mammalian palate (Neiswender et al., 2017). Loss of function of the Wnt modulator Sfrp3 in zebrafish results in the failure of anterior palate extension, further highlighting the role of Wnt signaling in palatal extension and convergence in zebrafish (Kamel et al., 2013).

### Chick models

Orofacial clefts have also been observed in chick embryos (Abramyan and Richman, 2018), where Wnt signaling similarly mediates the growth of primordial facial processes and the developing palate, in which six epithelial and three mesenchymal Wnt ligands, as well as several other pathway components, are expressed (Geetha-Loganathan et al., 2009). Wnt11 was shown to activate the non-canonical Wnt/PCP pathway and inhibit canonical Wnt/β-catenin signaling in the maxillary prominence, and its ectopic expression results in a notched beak/cleft lip phenotype (Geetha-Loganathan et al., 2014). Similarly, overexpression of Wnt2b leading to ectopic expression of msh homeobox 1 (Msx1) results in a foreshortened rostrum/upper beak, corresponding with a mammalian CLP phenotype (Medio et al., 2012).

### Frog models

Recently, the suitability of *Xenopus* embryos for transplanting tissue and local chemical perturbation have provided a suitable clefting model (Dickinson, 2016). Although several studies have assessed the involvement of various biochemical pathways and factors in frog palatal clefts, including retinoic acid and folate metabolism, few studies have probed Wnt signaling during orofacial development in this organism (Dickinson and Sive, 2009; Kennedy and Dickinson, 2012; Wahl et al., 2015).

### *In vitro* models

Another means by which investigators study secondary palate fusion is by culturing palatal shelf explants and assaying their ability to complete the final stages of palate fusion *in vitro*, such as adherence and formation of the MES and subsequent apoptosis to establish mesenchymal confluence (Ibrahim et al., 2015). Although not directly analogous to *in vivo* palatogenesis, this approach has helped examine the roles of many factors and processes that are important for the fusion process specifically, including Wnt11 and its dependence on Fgf signaling in palatal closure (Lee et al., 2008).

## Crosstalk between Wnt signaling, cell adhesion molecules and transcription factors in orofacial clefts

**Fig. 2.**
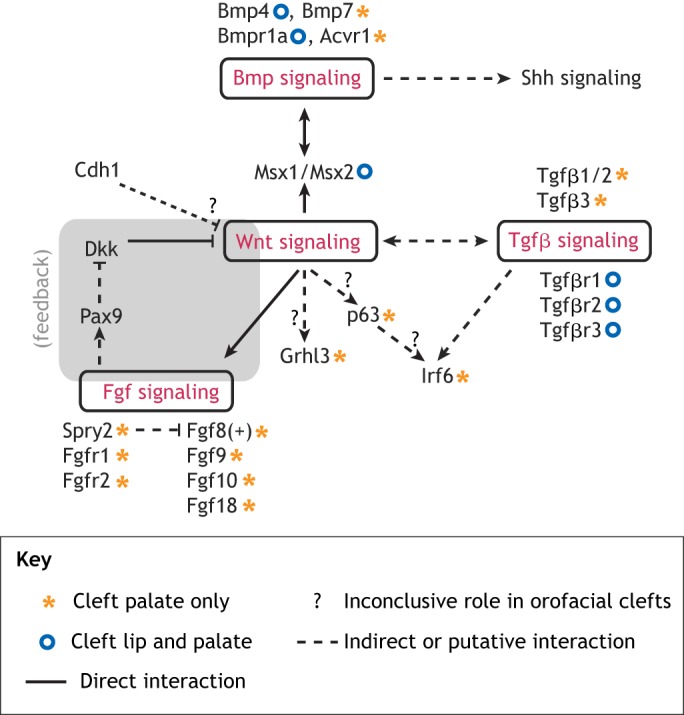
**Crosstalk between Wnt, Cdh1, Bmp, Tgfβ and Fgf signaling pathways in orofacial clefts.** In lip and palate primordia, the cell adhesion molecule Cdh1 may negatively regulate canonical Wnt signaling, which may interact with Bmp signaling through the common targets Msx1/Msx2. Wnt signaling also regulates Tgfβ signaling in palatogenesis. Tgfβ signaling modulates the orofacial cleft-causing gene *Irf6*, which may also be regulated by Wnt signaling through p63. Moreover, Wnt signaling can activate Fgf signaling, which modulates Pax9 to repress Dkk protein, an inhibitory ligand of Lrp6 in the canonical Wnt pathway, forming a positive-feedback regulatory loop during orofacial development. The Spry protein family is known to inhibit Fgf signaling. Grhl3 and Irf6 are well-known candidates for Van der Woude syndrome, a syndromic form of CLP. However, their regulation by Wnt has yet to be elucidated. The phenotypic outcomes of the key signaling components, demonstrated in mutant mouse models, are marked as per the figure key.

Because β-catenin has dual roles in Wnt signaling and in cell adhesion, it remains unclear which functions of β-catenin are required for which stages of orofacial development. The roles of other cell-cell adhesion proteins, such as E-cadherin (Cdh1), during palatogenesis remain to be elucidated (reviewed in Lough et al., 2017). Mutations in *CDH1* have been associated with an increased risk for non-syndromic orofacial clefts in humans (Rafighdoost et al., 2013; Vogelaar et al., 2013; Bureau et al., 2014; Hozyasz et al., 2014; Brito et al., 2015; Ittiwut et al., 2016; Song et al., 2017). In mouse models, *Cdh1* knockout is embryonic lethal and mutant embryos do not develop beyond E10.5 (Garcia-Higuera et al., 2008). Conditional *Cdh1* knockout in neural crest cells results in craniofacial defects related to bone development, including a shortened snout, abnormal teeth and twisted nasal bones (Shao et al., 2016). However, these mutants did not develop orofacial clefts. A possible interaction between CDH1 and the WNT signaling pathway has been suggested in human endometrial epithelial cells, where ablation of CDH1 enhances canonical WNT signaling (Zhu et al., 2018) (Fig. 2). Furthermore, increased expression of Cdh1 in mouse maxillary mesenchymal cells during palatogenesis results in a reduction of cytosolic β-catenin (Warner et al., 2016) (Table 2). These studies suggest that Cdh1 may negatively regulate the canonical Wnt/β-catenin signaling pathway in humans and mice (Table 2, Fig. 2).

**Table 2.**
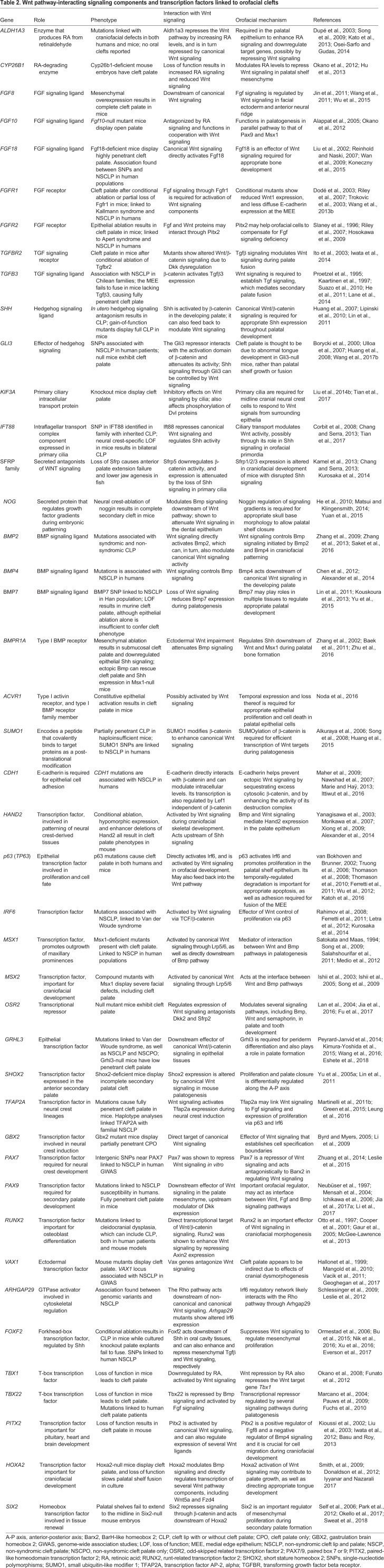
**Wnt pathway-interacting signaling components and transcription factors linked to orofacial clefts**

Individuals with mutations in either of the epithelial transcription factors grainyhead-like transcription factor 3 (GRHL3) and interferon regulatory factor 6 (IRF6), detected in families with Van der Woude syndrome (Box 1), tend to present with CLP (de Lima et al., 2009; Peyrard-Janvid et al., 2014). Further investigation in mice suggested that there is no epistatic interaction between these two transcription factors during palatogenesis (Peyrard-Janvid et al., 2014). However, both Grhl3 and Irf6 can be activated in mouse epithelial cells by Wnt/β-catenin signaling (Fig. 2), either directly or indirectly (Ferretti et al., 2011; Kimura-Yoshida et al., 2015). Therefore, Grhl3 and Irf6 may have essential roles during palatogenesis, acting in pathways that diverge from each other downstream of the canonical Wnt pathway and converge again during later stages of palatal development. Irf6 can directly activate Grhl3 (de la Garza et al., 2013), and Grhl3 expression is significantly reduced during embryonic development of Irf6-null mice (Fakhouri et al., 2017) (Table 2). Grhl3-null embryos die shortly after birth, presenting defects in skull morphology (Ting et al., 2005; Goldie et al., 2016). During early embryogenesis, Grhl3 expression is restricted to ectodermal and epidermal lineages (Wang and Samakovlis, 2012; Peyrard-Janvid et al., 2014). The importance of the grainyhead-like gene family, which includes *Grhl3*, is emphasized by its high level of conservation between fungi and animals, and its conserved role in epithelial specification (Paré et al., 2012; Miles et al., 2017). Exome sequencing-based association studies have revealed several variations in human *GRHL3* associated with non-syndromic CPO in almost all populations studied (Leslie et al., 2016; Mangold et al., 2016; Hoebel et al., 2017; Eshete et al., 2018) (Table 2), except in the Han Chinese (He and Bian, 2016), suggesting *GRHL3* as a strong candidate gene for non–syndromic CPO. Thus, proper Grhl3 expression in the epithelium during palatogenesis seems to be crucial for normal palate formation (Carpinelli et al., 2017). In mice, a cooperative interaction has been suggested between Grhl2 and Grhl3 during primary neurulation (Box 1) (Rifat et al., 2010), and Grhl3 might act downstream of canonical Wnt signaling during neural tube closure (Kimura-Yoshida et al., 2015). Nevertheless, it remains unclear whether Grhl3 is a direct downstream target of the canonical Wnt/β-catenin signaling, or whether Grhl2 and Grhl3 act cooperatively during palate development.

Analysis of Irf6 expression and function in mouse and chick developmental models suggests that Irf6 might play a role in tissue fusion events during palatogenesis (Knight et al., 2006; Velazquez-Aragon et al., 2016). The Wnt target p63 (also known as TP63), a key regulator of proliferation and differentiation (Truong et al., 2006), inhibits the Wnt signaling output by repressing Wnt/β-catenin responsive elements in target genes (Katoh et al., 2016). p63 may directly activate Irf6 in the facial ectoderm, and a defective pre-B cell leukemia homeobox (Pbx)-Wnt-p63-Irf6 signaling cascade has been suggested in cleft lip formation (Ferretti et al., 2011). Irf6 interacts upstream of the cleft-associated Rho GTPase-activating protein 29 (Arhgap29), which regulates Rho activity downstream of Wnt5a in the PCP pathway (Leslie et al., 2012). Furthermore, several genome-wide association studies have uncovered interactions between *IRF6* and other factors linked with orofacial cleft susceptibility. Li and colleagues have identified a three-way gene interaction of SNPs in *IRF6*, *WNT5A* and *C1orf107* (also known as *UTP25*, a nucleolar protein), and a separate interaction between *IRF6* and *WNT2*, in association with NSCLP (Li et al., 2015). *IRF6* mutations were also associated with SNPs in the actin-binding protein tropomysin (*TPM1*) and in the axon guidance signaling molecule netrin 1 (*NTN1*) (Velazquez-Aragon et al., 2016).

## Wnt signaling crosstalk with other morphogenetic signaling pathways in orofacial clefts

Wnt signaling does not act in isolation during lip/palate development. Several other signaling pathways are involved in orofacial development, including the fibroblast growth factor (Fgf), bone morphogenic protein (Bmp), transforming growth factor beta (Tgfβ), sonic hedgehog (Shh) and retinoic acid (RA) signaling pathways (Iwata et al., 2011; Bush and Jiang, 2012; Cobourne and Green, 2012; Parada and Chai, 2012; Stanier and Pauws, 2012; Wang et al., 2013; Kurosaka et al., 2014; Okano et al., 2014; Yan et al., 2018; Graf et al., 2016;). This section discusses how these pathways interact with each other and with Wnt signaling.

### Wnt-Fgf signaling crosstalk

Wnt/β-catenin signaling activates *Fgf8* expression in early facial patterning (Wang et al., 2011), while Fgf8 induces paired box 9 (*Pax9*) expression during palatogenesis (Neübuser et al., 1997) (Fig. 2). Fgf8 overexpression in mice results in cleft palate, while Fgf10, a loss of which also results in cleft palate, seems to function in cooperation with Wnt signaling (Alappat et al., 2005; Wu et al., 2015). Knockout of other Fgf genes (*Fgf9* and *Fgf18*) and their receptors (Fgfr1 and Fgfr2) has also been associated with a cleft palate phenotype (Liu et al., 2002; Trokovic et al., 2003; Rice et al., 2004) (Fig. 2). Pax9 may feed back into and regulate canonical Wnt/β-catenin signaling in the anterior palatal mesenchyme during palatogenesis. Pax9 ablation causes an increase of the Wnt signaling modulators dickkopf Wnt signaling inhibitor 1 and 2 (Dkk1 and Dkk2), and intravenous delivery of small-molecule Dkk inhibitors can rescue the cleft palate phenotype *in utero* in Pax9-null mouse embryos (Jia et al., 2017b; Li et al., 2017) (Fig. 2). Likewise, Pax9 seems to target Wnt signaling through the downregulation of β-catenin and Axin2 in the canonical Wnt signaling pathway (Li et al., 2017). Knockout of the human cleft palate-associated transcription factor Tbx22 in mice results in submucous CPO and ankyloglossia (Marçano et al., 2004; Pauws et al., 2009). Tbx22 is at the interface between Bmp and Fgf signaling, being repressed by the former and activated by the latter, downstream of canonical Wnt signaling in the developing palate (Fuchs et al., 2010). Sprouty 2 (*Spry2*) is also a candidate Wnt/β-catenin target gene (Ordόñez-Morán et al., 2014), and Spry family members inhibit Fgf signaling. Spry2 knockout mice display aberrant growth and movement of palatal shelves, and cleft palate occurs due to a failure of palatal shelf elevation (Welsh et al., 2007; Matsumura et al., 2011) (Fig. 2). These animals show altered expression of the Bmp target and cleft palate gene *Msx1*, implying a possible link between Fgf and Bmp signaling in the palate (Welsh et al., 2007). Moreover, Spry4 signaling interacts with the Irf6 pathway, which, as we discuss above, is a crucial player in orofacial development (Kousa et al., 2017).

### Wnt-Bmp-Shh signaling crosstalk

The homeobox-containing Msx transcription factors function as downstream effectors of Bmp signaling in many developmental processes, including in palatogenesis (Cheng et al., 2003; Tribulo et al., 2003; Hayashi et al., 2006; Parada and Chai, 2012). Bmp signaling directly activates Msx genes at early stages of ectodermal patterning in order to specify the neural crest (Graham et al., 1994; Tribulo et al., 2003). Lrp6-mediated Wnt signaling also regulates *Msx1* and *Msx2* expression in the orofacial primordia, but Lrp6 ablation does not affect Bmp4 during primary lip/palate formation and fusion, suggesting that the Bmp and Wnt pathways may converge by way of common activation of Msx1/Msx2 (Song et al., 2009) (Fig. 2). Mice with knockouts of either Msx1 or the Bmp receptor Bmpr1a display cleft palate and downregulated Shh signaling. Bmp4 expression in the anterior palate mesenchyme is lost in Msx1-null mice, while transgenic ectopically expressed human BMP4 is able to rescue both Shh activity and the cleft palate phenotype in these animals (Zhang et al., 2002; Baek et al., 2011). This implies that Bmp proteins are the primary effectors of Msx1 activity, and that an Msx1-Bmp-Shh cascade may act downsteam of Lrp6-mediated Wnt signaling to regulate palatogenesis. This link between Bmp and Shh signaling in the palate primordia appears to be mediated by the epithelial transcription factor heart and neural crest derivatives expressed 2 (Hand2) (Xiong et al., 2009).

Many Bmp receptors are expressed in differing patterns along the anterior-posterior (A-P) axis of the palatal shelves during palatogenesis. Submucous cleft palate (Box 1) results from the overexpression of the Bmp receptor activin A receptor-type I (Acvr1) (Noda et al., 2016), so appropriate levels and localization of Bmp pathway activity appear critical for correct tissue responses to palatogenic signaling. Conditional deletion of the Bmp signaling receptors Bmpr1a or Acvr1 in neural crest cells results in multiple craniofacial abnormalities, including submucous cleft palate (Dudas et al., 2004; Saito et al., 2012) (Fig. 2). Interestingly, Wnt9b may regulate Bmp4 during lip fusion (Lan et al., 2006), but this interaction has not been demonstrated during palate fusion. Wnt5a, however, is expressed in a descending gradient from the anterior to the posterior developing palate, and it can act as a negative regulator of Bmp4 in a concentration-dependent manner across the palate (He et al., 2008). Bmp2 seems unaffected by Wnt5a, thus Bmp2 activation may occur in a separate pathway from that of Bmp4 (He et al., 2008). Bmp7 is also expressed in the developing palate and during rugae (Box 1) formation, where it acts downstream of canonical Wnt signaling (Lin et al., 2011), and has been linked with cleft palate in both humans and mice (Kouskoura et al., 2013; Yu et al., 2015). Bmp signaling is upregulated in homeobox A2 (Hoxa2)-null embryos, and Hoxa2 may inhibit palatal osteogenic differentiation from mesenchymal cells via its modulation of Bmp signaling (Iyyanar and Nazarali, 2017). In addition, homeobox protein sine oculis-related homeobox 2 (Six2) likely acts as a downstream effector of Hoxa2 in regulating mesenchymal cell proliferation during secondary palate formation (Okello et al., 2017), and palatal shelves fail to extend to the midline in Six2 knockout mice (Sweat et al., 2018). It remains unclear whether this activity is related to the interaction of Hoxa2 with Bmp. Six2 is known to repress Wnt/β-catenin by binding to T-cell factor/lymphoid enhancer binding factor 1 (Tcf/Lef1) family members during nephrogenesis (Self et al., 2006; Park et al., 2012), but it remains unclear whether Six2 does so during palatogenesis.

### Wnt-Tgfβ signaling crosstalk

**Fig. 3.**
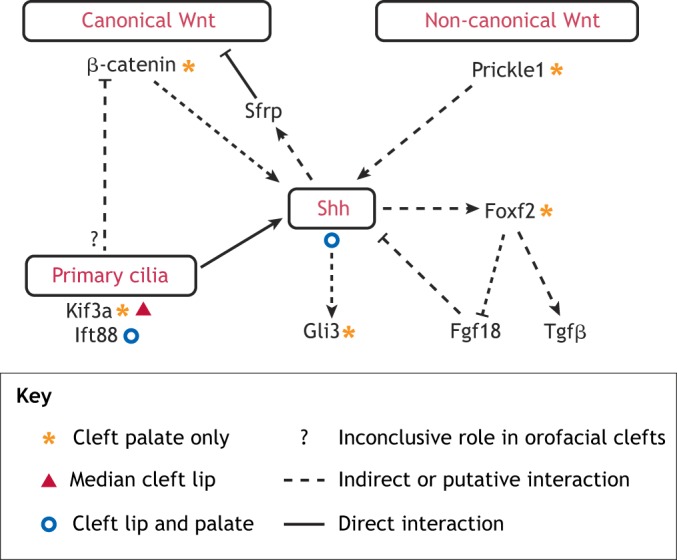
**Crosstalk between Wnt, Shh, primary cilia and other related signaling components in orofacial clefts.** Shh signaling, which is regulated by primary cilia or intraflagellar transport proteins, represses canonical Wnt signaling through the negative Wnt regulator Sfrp, while β-catenin may regulate Shh signaling, suggesting a negative-feedback loop between the Wnt and Shh pathways in lip/palate formation. The non-canonical Wnt signaling molecule Prickle1 also activates Shh signaling, which may subsequently inhibit the canonical Wnt pathway. There is a complex interplay between the Fox, Fgf, Shh and Tgfβ family members during lip/palate development. The phenotypic outcomes of the key signaling components highlighted in this figure were demonstrated in the respective mutant mouse models.

Epithelial Wnt/β-catenin signaling also regulates Tgfβ signaling. Wnt-mediated Tgfβ3 activation is required for MEE cell apoptosis during palatal shelf closure (He et al., 2011) (Fig. 2), and knockout of all three isoforms of Tgfβ has been associated with cleft palate in mice, in either single or doubly mutant lines (Kaartinen et al., 1995; Sanford et al., 1997; Jin and Ding, 2014) (Fig. 2). Tgfβ1 and Tgfβ3 are semi-redundant, and overexpression of Tgfβ1 can partially rescue the cleft phenotype observed in Tgfβ3-null mice (Yang and Kaartinen, 2007). Mutations in transforming growth factor beta receptor 3 (Tgfβr3, also known as betaglycan), which binds Tgfβ ligands without transducing the signal, cause cleft palate due to reduced cell proliferation and increased apoptosis (Hill et al., 2015) (Fig. 2). By contrast, conditional Tgfβr1 and Tgfβr2 knockout in neural crest cells also causes cleft palate and skull defects due to insufficient cell proliferation (Ito et al., 2003; Dudas et al., 2006) (Table 2, Fig. 2). Tgfβ signaling through epithelial Tgfβr2 feeds back into the Wnt pathway by repressing Dkk1 and Dkk4 to enhance mesenchymal Wnt signaling activity (Iwata et al., 2014). The forkhead box transcription factor Foxf2, which represses Wnt signaling in the gastrointestinal system (Ormestad et al., 2006), may effect Tgfβ signaling during palate development, and has been linked to orofacial clefts in both mice and humans (Bu et al., 2015; Nik et al., 2016) (Table 2, Fig. 3). Foxf2 ablation downregulates Tgfβ2 during palatogenesis, causing a decrease in mesenchymal cell proliferation and aberrant collagen accumulation (Nik et al., 2016), resulting in cleft palate in mice (Wang et al., 2003) (Table 2, Fig. 3). Repression of Wnt signaling by Foxf2 has been demonstrated in intestinal fibroblasts (Nik et al., 2013), although a direct relationship between Foxf2 and Wnt signaling during palatogenesis remains undemonstrated.

### Wnt-Shh-cilia crosstalk

Hedgehog signaling during embryogenesis depends on primary cilia function and intraflagellar transport (Huangfu et al., 2003; Huangfu and Anderson, 2005) (Fig. 3). Individuals with ciliopathies resulting from defects of the primary cilia often have CLP, and tissue-specific deletion of the intraflagellar transport genes intraflagellar transport 88 (*Ift88*) or kinesin family member 3A (*Kif3a*) in mice causes CLP (Liu et al., 2014b; Schock et al., 2017; Tian et al., 2017) (Fig. 3). Mouse pups with conditional deletion of Ift88 in cranial neural crest cells with Wnt1-driven Cre die at birth due to severe craniofacial defects, including bilateral CLP (Box 1), whereas elimination of Ift88 specifically in the palatal mesenchyme results in CPO (Tian et al., 2017). Loss of Ift88 results in a downregulation of Shh signaling in the palatal mesenchyme (Tian et al., 2017). In addition, a novel missense mutation in *IFT88* has been reported in a family affected by isolated CLP, suggesting it as a candidate gene for orofacial clefts (Tian et al., 2017). Both Ift88 and Kif3a may repress canonical Wnt signaling (Corbit et al., 2008; Chang and Serra, 2013) (Fig. 3). Combined, these results underscore the significance of intraflagellar proteins in craniofacial development, which involves Shh signaling, and the role of Shh signaling in feeding back to negatively regulate Wnt signaling.

The ventral anterior homeobox (Vax) transcription factors are important for neural patterning, and they mediate signaling between Shh and Wnt (Vacik et al., 2011). Sfrp and ventral anterior homeobox 1 (Vax1) are the downstream effectors of Shh signaling, and Shh, in turn, inhibits Wnt/β-catenin signaling (Kurosaka et al., 2014) (Fig. 3). Furthermore, *VAX1* is a candidate human NSCLP gene (Mangold et al., 2010), and Vax1-null mouse embryos exhibit cell proliferation problems during cranial development around E10.5, possibly due to a downregulation of Shh. These embryos do not present with a CLP phenotype, suggesting that Vax1 does not play a direct role in palatogenesis (Geoghegan et al., 2017). Palatal rugae are established by Shh expression, which is opposed by Fgf signaling at the inter-rugal regions of the epithelium (Economou et al., 2012). Wnt/β-catenin signaling is also required for Shh induction in the palatal rugae (Lin et al., 2011; Kawasaki et al., 2018).

Wnt5a/Ror2 may act upstream of the non-canonical Wnt signaling molecule Prickle1 (Liu et al., 2014a; Yang et al., 2014) (Fig. 1), and Prickle1 itself may act upstream of Shh during palatogenesis (Yang et al., 2014) (Table 2, Fig. 3). In osteoblast-lineage cells, the non-canonical and canonical Wnt pathways have a positive relationship, and Wnt5a upregulates Wnt/β-catenin signaling, while its ablation inhibits canonical signaling by reducing Lrp5/Lrp6 expression (Okamoto et al., 2014). Wnt5a may also act upstream of Msx1, Bmp2, Bmp4 and Shh during palatogenesis, placing Wnt5a as a promising candidate for signaling pathway crosstalk during palate development (He et al., 2008; Smith et al., 2012). Although Msx1 expression was downregulated in Wnt5a knockout mouse palates, an Msx1-binding enhancer was identified upstream of Wnt5a, implying a possible synergistic relationship between these two factors (He et al., 2008; Nishihara et al., 2016).

Shh has been reported to activate Fox genes during lip and palate development (Jeong et al., 2004; Nik et al., 2016; Everson et al., 2017). Foxf2 also represses Fgf18 signaling from the palatal mesenchyme, which itself negatively regulates Shh expression in the palatal epithelium, leading to reduced Shh expression in Foxf2 knockout mice (Xu et al., 2016), which suggests a positive-feedback loop (Fig. 3). Gli3 acts as an activator of hedgehog pathway targets in the presence of Shh signaling and becomes a repressor when Shh signaling is absent (Wang et al., 2000). Moreover, GLI3 has been associated with NSCLP in human patients (Wang et al., 2017b), and Gli3-null mouse embryos exhibit cleft palate and tongue abnormalities due to improper tongue morphogenesis and failure of palatal shelf elevation and fusion (Table 2, Fig. 3) (Huang et al., 2008). The repressor form of Gli3 modulates Wnt signaling and physically interacts with β-catenin, linking the Shh and Wnt pathways (Ulloa et al., 2007).

### Wnt-RA-Fgf signaling crosstalk

**Fig. 4.**
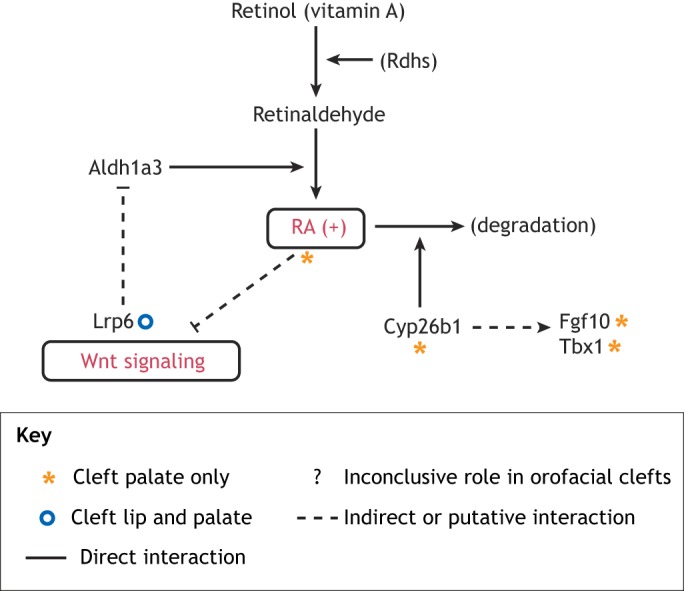
**Crosstalk between Wnt, RA and related signaling pathways in orofacial clefts.** A surplus (+) of RA induces cleft palate, and RA is known to interact directly with the canonical Wnt pathway. In orofacial primordia, retinol dehydrogenases (Rdhs) convert vitamin A (retinol) to retinaldehyde, which subsequently produces RA mainly via the aldehyde dehydrogenase Aldh1a3. RA represses Wnt signaling, while Lrp6-mediated canonical Wnt signaling represses Aldh1a3 expression in the orofacial epithelium, suggesting a reciprocal negative regulation between the Wnt and RA pathways. The cytochrome P450 family member Cyp26b1 is responsible for RA degradation, thereby regulating the endogenous levels of RA. Fgf10 and Tbx1 may act downstream of Cyp26b1 during palatogenesis. The phenotypic outcomes of the key signaling components highlighted in this figure were demonstrated in the respective mutant mouse models.

RA plays an important role in normal palatogenesis (Okano et al., 2014), and excess RA exposure in human and murine embryos can cause orofacial clefts (Abbott and Pratt, 1987; Abbott et al., 1989). Several aldehyde dehydrogenases are involved in the synthesis of RA from retinaldehyde, with aldehyde dehydrogenase family 1, subfamily A3 (Aldh1a3) being largely responsible for RA production in the oral epithelium (Kato et al., 2013). RA signaling interacts with the Wnt/β-catenin pathway (Fig. 4) (Kumar and Duester, 2010; Yasuhara et al., 2010; von Gise et al., 2011; Zhao and Duester, 2009; Osei-Sarfo and Gudas, 2014), and alters cellular proliferation and apoptosis in the craniofacial mesenchyme and epithelium through its repression of Wnt signaling in palatogenesis (Hu et al., 2013). Canonical Wnt signaling appears to feed back into and inhibit RA signaling, as Aldh1a3 is ectopically expressed in the upper lip primordia of Lrp6-deficient embryos (Song et al., 2009) (Fig. 4). Cytochrome P450, family 26, subfamily b, polypeptide 1 (Cyp26b1), the enzyme that degrades RA and therefore regulates endogenous RA levels, is required for proper elevation of palatal shelves, and Cyp26b1 knockout mice display cleft palate due to excess RA (Okano et al., 2012). Cyp26b1 enhances T-box 1 (Tbx1) and Fgf10 expression in the oral epithelium, while an excess of RA represses both. Fgf10 expression is lost in Cyp26b1-null mice, and palatal Tbx1 expression was downregulated when murine fetuses were treated with exogenous RA (Okano et al., 2008; Okano et al., 2012) (Fig. 4). Both Tbx1-null and Fgf10-null mice display cleft palate (Alappat et al., 2005; Funato et al., 2012), suggesting that these important regulators of palatal shelf elevation act downstream of Cyp26b1, and their expression is likely modulated by RA levels. As researchers continue to identify the factors that connect these different pathways, it becomes increasingly important to understand how they are regulated.

### Crosstalk of Wnt signaling with epigenetic regulators in orofacial clefts

Sequence-independent gene regulatory mechanisms, such as histone modification, DNA methylation and microRNA (miRNA) transcript regulation, have garnered increasing attention in recent years. These epigenetic mechanisms play a role in regulating many Wnt pathway components (Wils and Bijlsma, 2018). Studies suggest that miRNAs are involved in regulating Wnt signaling during palatogenesis; in mice, conditional deletion of Dicer1, the key effector of RNA interference (RNAi)-mediated mRNA cleavage, leads to craniofacial defects, including cleft palate (Zehir et al., 2010). A 2016 study of plasma miRNAs expressed in human NSCLP patients suggests that many key targets of dysregulated miRNAs share functional relationships with Wnt, Notch, hedgehog and lipid signaling pathways (Li et al., 2016). The miRNAs hsa-miR-24-3p, hsa-miR-1260b and hsa-miR-205-5p have been identified in a human transcriptome screen as candidates for NSCLP, and were computationally predicted to target several Wnt signaling pathway components (Wang et al., 2017a). Another miRNA, miR-544a, has been associated with downregulation of *CDH1* during EMT in cancer cells, in turn activating the Wnt signaling pathway (Yanaka et al., 2015). The miR-17-92 cluster reportedly targets transcripts of NSCLP-associated Wnt target genes *Tbx1* and *Tbx3*, and is itself a target of Bmp signaling and of the craniofacial pioneer factor AP-2α. miR-17-92 knockout in mouse embryos results in severe craniofacial defects, including CLP, the severity and penetrance of which are increased in miR-17-92;miR-106b-25 compound mutants (Wang et al., 2013a). In zebrafish, platelet-derived growth factor (Pdgf) signaling is an important regulator of palatogenesis, and it is modulated by miR-140 during palatogenesis (Eberhart et al., 2008). Further studies discerning whether the described roles of miRNAs in other cellular processes resemble their roles in palatogenesis may contribute to our understanding of the mechanisms by which cleft palate arises.

Several studies examined epigenetic modifications in NSCLP, including DNA methylation (Alvizi et al., 2017; Sharp et al., 2017). The transcription factor and human CLP candidate SOX4, the targets of which include *FZD5* (Scharer et al., 2009), has been implicated in studies of genomic regions that are differentially methylated during palatogenesis (Seelan et al., 2012). Changes in *CDH1* promoter methylation levels in human blood and lip tissue have been correlated with NSCLP, as well as with differences in NSCLP penetrance in susceptible families, implying that DNA methylation patterns may account for the variable penetrance of CLP phenotypes (Alvizi et al., 2017). A stochastic deficiency in DNA methylation of a retrotransposon near the coding region of *Wnt9b* in the A/WySn mouse impaired its transcription and contributed to an incompletely penetrant CLP phenotype (Juriloff et al., 2014). The role of histone modifications in craniofacial development is less understood, but recent studies suggest that histone H3 acetylation can play a role in the formation of cleft palate in mouse due to dysregulation of Tgfβ signaling, although how this process affects Wnt signaling has not been demonstrated (Yuan et al., 2016). However, the histone acetyltransferase p300 (also known as Ep300) is important for gene regulation, and its ablation in mouse palatal mesenchyme cells results in altered Wnt signaling, as well as in aberrant Wnt-dependent proliferation and migration (Warner et al., 2016). An improved understanding of epigenetic regulation of Wnt signaling and related pathways may hold the key to addressing the impact of environmental and non-genetic factors on the presentation of orofacial clefts.

## Translational perspectives

To date, the most prevalent treatment for orofacial clefts is surgical repair coupled with nasoalveolar molding to direct postnatal tissue growth and subsequent orthodontic treatment (Chen et al., 2005). Protocols and procedures have varied widely, not only in developing areas of the world, but within developed countries as well (Mossey et al., 2009). A better understanding of the complex interactions between components of the Wnt and other signaling pathways that govern lip/palate formation will provide better opportunities for treatment and prevention of orofacial clefts through cellular- and molecular-based methods, reducing the need for surgical intervention (Panetta et al., 2008). Research in animal models has identified several altered pathways that, when targeted, reversed orofacial clefts. Direct modulation of Wnt signaling by chemically stabilizing a catalytically inactive allele of the canonical Wnt pathway factor Gsk3 has shown therapeutic potential in mice, where its timely reactivation could reverse a cleft palate phenotype in Gsk3β-deficient mice (Liu et al., 2007). Additionally, ectopic expression of Wnt in the ectoderm rescued the orofacial cleft phenotype in Pbx-deficient mouse models (Feretti et al., 2011). Modulation of Shh signaling has also been shown to rescue cleft palate in the Msx1-null mouse model, both through ectopic Bmp expression and through downregulation of distal-less homeobox 5 (Dlx5) (Zhang et al., 2002; Han et al., 2009). Reynolds and colleagues have shown that administration of either 3-4 mg/kg folic acid or 140-187 mg/kg methionine to pregnant mice that were previously treated with intraperitoneal RA to induce CLP reduces the frequency of cleft palate to 6%, compared with 76% in RA-treated controls. Interestingly, the combined folic acid and methionine treatment completely rescued the RA-induced aberrant palatogenesis (Reynolds et al., 2003).

Utilizing controlled intravenous delivery of the small-molecule Wnt agonists WAY-262611 and IIIc3a (both acting as Dkk inhibitors) into *Pax9* mutant mice rescues the growth and fusion of palatal shelves by restoring Wnt signaling (Jia et al., 2017a; Li et al., 2017). In addition to small-molecule modulation, synthetic ligand analogs have also shown potential to stimulate Wnt signaling (Andersson et al., 2015; Zhan et al., 2017) and could lead to the development of future treatments for orofacial clefts. Additionally, genetic inactivation of Wise (also known as Sostdc1), a canonical Wnt antagonist, in Pax9-deficient mouse embryos rescued the palatal shelf elevation, mainly through restoring hyaluronic acid accumulation in the palatal mesenchyme (Li et al., 2017). Wnt5a analogs, such as Foxy-5 or Box-5, currently used in cancer research (Andersson et al., 2015; Zhan et al., 2017), may also serve as a treatment approach for orofacial clefts by targeting non-canonical Wnt signaling, warranting future research. Taken together, these reports indicate that Wnt signaling modulators could contribute to an effective molecular treatment regime for orofacial clefts.

Given the heterogeneous causes of orofacial clefts and the variability in the genotypes of affected individuals, it is unlikely that we will see a ‘one-size’ approach to non-surgical orofacial cleft treatment any time soon. However, successful studies using mutant mouse models are promising for the prospect of pathway-specific treatments to allow for prenatal intervention when a fetal genotype renders human embryos at risk of orofacial cleft development. Implementation of promising therapies to human patients would impinge on the timing of application and on the accurate detection of improper palatogenesis. Even if parents carrying alleles linked to orofacial clefts were to commit to prenatal genotyping, many palatal development processes occur early in gestation. Even with improved protocols for correcting the levels of a target signaling factor in patients, implementing postconception measures at such an early stage of pregnancy, before many mothers know that they are pregnant, is challenging. Further challenges arise from the high variability in the manifestation and penetrance of orofacial cleft phenotypes. The complexity of the molecular processes that govern orofacial development makes it difficult to predict the therapeutic requirement. Moreover, a particular intervention may not be applicable to more than a minor subset of cases, even in individuals with defects in a particular gene or pathway. A deeper understanding of the pathways that govern palatogenesis may allow *in vitro* fertilization with selected gametes that do not possess the risk-imparting allele.

Interactions between Wnt/β-catenin signaling and other morphogenetic signaling pathways are widely employed in many different developmental programs. The nature of embryonic development creates significant potential for off-target effects and disruption of other essential developmental processes in both mother and child if molecular treatments to correct palatogenesis errors are applied systemically. Before treatments targeting signaling pathways could be considered for clinical trials with human patients, such risks would need to be thoroughly explored and addressed, and likely require new delivery techniques more advanced than those currently available. Additionally, because NSCLP is not life threatening, many parents may be unwilling to attempt untested and potentially dangerous approaches, despite the burden and difficulty of current treatments. However, in the future, a more complete understanding of morphogenetic pathway crosstalk and the systemic impact of perturbations to them may eventually allow the progression of molecular clinical approaches to a point at which they are considered safe. A robust understanding of how signaling pathways function in all systems and processes during development will be able to not only inform studies related to orofacial clefts, but also contribute to the development of treatments for other syndromes and disorders with Wnt pathway etiologies. Despite the many barriers that still remain, knowledge of developmental mechanisms is helping to, and will continue to, facilitate the refinement of techniques for the application of that knowledge to develop the means to safely and effectively treat congenital disorders like orofacial clefts.
